# Fetal Heart Rate Fragmentation

**DOI:** 10.3389/fped.2021.662101

**Published:** 2021-09-01

**Authors:** Matilde Costa, Mariana Xavier, Inês Nunes, Teresa S. Henriques

**Affiliations:** ^1^Department of Biomedical Engineering, Faculty of Engineering, Universidade do Porto, Porto, Portugal; ^2^Centro Materno-Infantil do Norte, Centro Hospitalar e Universitário do Porto, Porto, Portugal; ^3^Centre for Health Technology and Services Research (CINTESIS), Faculty of Medicine University of Porto, Porto, Portugal; ^4^ICBAS School of Medicine and Biomedical Sciences, University of Porto, Porto, Portugal; ^5^Department of Health Information and Decision Sciences-MEDCIDS, Faculty of Medicine, University of Porto, Porto, Portugal

**Keywords:** fetal heart rate, fragmentation, symbolic dynamics, short-term variability, acidemia, umbilical cord pH

## Abstract

Intrapartum fetal monitoring's primary goal is to avoid adverse perinatal outcomes related to hypoxia/acidosis without increasing unnecessary interventions. Recently, a set of indices were proposed as new biomarkers to analyze heart rate (HR), termed HR fragmentation (HRF). In this work, the HRF indices were applied to intrapartum fetal heart rate (FHR) traces to evaluate fetal acidemia. The fragmentation method produces four indices: PIP-Percentage of inflection points; IALS-Inverse of the average length of acceleration/deceleration segments; PSS-Percentage of short segments; PAS-Percentage of alternating segments. On the other hand, the symbolic approach studied the existence of different patterns of length four. We applied the measures to 246 selected FHR recordings sampled at 4 and 2 Hz, where 39 presented umbilical artery's pH ≤ 7.15. When applied to the 4 Hz FHR, the PIP, IASL, and PSS showed significantly higher values in the traces from acidemic fetuses. In comparison, the percentage of “words” $W_{1}^{h}$ and $W_{2}^{s}$ showed lower values for those traces. Furthermore, when using the 2 Hz, only IASL, *W*_0_, and $W_{2}^{m}$ achieved significant differences between traces from both acidemic and normal fetuses. Notwithstanding, the ideal sampling frequency is yet to be established. The fragmentation indices correlated with Sisporto variability measures, especially short-term variability. Accordingly, the fragmentation indices seem to be able to detect pathological patterns in FHR tracings. These indices have the advantage of being suitable and straightforward to apply in real-time analysis. Future studies should combine these indexes with others used successfully to detect fetal hypoxia, improving the power of discrimination in a larger dataset.

## 1. Introduction

In the twentieth century, technical advances led to the development of continuous electronic monitoring of fetal heart rate (FHR) and uterine contraction (UC) signals, a technology known as cardiotocography (CTG) (1). This technology constitutes the primary screening method to allow early recognition of fetal distress related to intrapartum fetal hypoxia/ acidosis. Intrapartum fetal monitoring's principal goal is to avoid adverse perinatal outcomes related to hypoxia/acidosis without causing an increase in unnecessary obstetrical interventions, such as cesarean sections or instrumental vaginal deliveries, which are associated with higher maternal and perinatal risks perinatal (2). Intrapartum fetal hypoxia is associated with the lack of an adequate oxygen supply to the fetus, which may lead to metabolic acidosis that, if not reversed, may cause cell dysfunction and death. The involvement of important fetal organs and systems may cause permanent sequelae, such as hypoxic-ischemic encephalopathy (HIE) in the short-term and cerebral palsy in the long-term or perinatal death. Before labor, on average, the arterial pH of a healthy fetus is around 7.35, whereas, at birth, the average pH of the umbilical artery blood is around 7.25. In this sense, it is considered that moderate neonatal acidosis/acidemia will occur when the pH is, at least, below 7.15 (1).

CTG became widely disseminated in industrialized countries, despite controversial scientific evidence in favor of its routine employment (1). The resulting graph is complex in nature and challenging to interpret. Considerable intra- and interobserver disagreement have been demonstrated in its analysis (3–5), both by inexperienced and experienced healthcare professionals (6–8), which limit CTG sensitivity and specificity. Computer analysis of CTGs was developed to overcome the poor inter and intraobserver agreement on tracing interpretation, to provide an objective evaluation of CTG features that are difficult to assess visually, and also to allow objective quantification of variability (9–11), a parameter that is closely related to the state of fetal oxygenation (12). There are different systems currently available that use different mathematical algorithms to elicit real-time alerts when changes associated with fetal hypoxia are detected (13, 14). Therefore, this is an adjunctive technology to CTG that aims to aid clinicians in the labor ward practice to intervene on time in order to avoid adverse perinatal outcomes related with hypoxia.

Commercially available FHR monitors acquire from Doppler or electrocardiographic signals, beat-to-beat intervals measured in milliseconds, and then convert and round off these values to provide a sequence of instantaneous FHRs, expressed in beats per minute (bpm) (15–17). When data is then exported from the FHR monitors to other devices, it is sampled at 4 Hz (there is an interpolation of signals so that an instantaneous FHR value is provided every 0.25 s) (15–17). Previous studies showed that while the linear time-domain parameters obtained from traces acquired at 2 or 4 Hz are correlated, the similar is not verified when using variability indices and nonlinear parameters, such as entropy (17, 18). In Romagnoli et al. (19) the authors compare several indices from 4 Hz traces and the corresponding down-sampled at 2, 1, 0.4, and 0.2 Hz. A better performance was obtained when using 2 Hz signals.

Recently, Costa et al. (20) proposed a new approach to analyze the heartbeat fragmentation to measure the short-term heart rate variability (STV). The assumption was that pathologic systems manifest the highest degree of heart rate fragmentation. The authors showed that these indices successfully distinguished the heartbeat of normal subjects from those with coronary artery disease. Furthermore, in a subsequent study, Costa et al. (21) introduced a similar approach to the previous analysis but using symbolic dynamics in order to get additional information on the temporal structure of heart rate fragmentation. Modanlou et al., in their study (22), observed that the STV was reduced along with neonatal hypoxemia, while more severe hypoxemia leads to the loss of long–term variability. On the other hand, Druzen et al. (23) showed that fetal hypoxia's early effects increased short and long term variability.

In this work, the new indices of both fragmentation methods were applied to FHR intrapartum traces to detect acidemia, comparing the traces sampling at 4 and 2 Hz.

## 2. Materials and Methods

### 2.1. Data

The database used in this work is available at Physionet (24)—*CTU-UHB Intrapartum Cardiotocography Database* (25). It contains 552 cardiotocography (CTG) intrapartum recordings with a maximum duration of 90 min each. For this work, it was only selected the last hour of the FHR recordings where the signal loss was lower than 15%. From the 246 selected recordings sampled at 4 Hz, 39 presented the umbilical artery's pH ≤ 7.15, which were considered cases of fetal acidemia (pathological). The 2 Hz traces were created, ignoring every other beat of 4 Hz sampling. The main clinical characteristics of the database are summarized in Table 1.

**Table 1:** Patient and labor characteristics for the CTU-UHB database (25).

**med [Q1, Q3]**	**Normal (*n* = 207)**	**Pathologic (*n* = 39)**	**Mann-Whitney *U*-test *P*-value**	**Cliff's delta effect size**
Gestational age (weeks)	39 [40, 41]	40 [41, 41]	0.007	–0.26 (s)
Weight (grams)	3,075 [3,370, 3,625]	3,225 [3,390, 3,650]	0.336	
Mother age	27 [30, 33]	26 [28, 30]	0.068	0.18 (s)
**n (%)**			**Fisher Test** ***P*** **-value**
Sex (female)	102 (49%)	19 (49%)	1
Diabetes	18 (9%)	1 (3%)	0.324
Hypertension	20 (10%)	2 (5%)	0.543
Preeclampsia	11 (5%)	1 (3%)	0.697
Pyrexia	4 (2%)	0	1
Meconium stained fluid	29 (14%)	4 (10%)	0.797
Induced labor	95 (46%)	14 (36%)	0.293
Vaginal delivery	207 (100%)	38 (97%)	0.159

*med, median; Q1, first quartile; Q3, third quartile. s, small effect size*.

### 2.2. SisPorto

The Omniview-SisPorto system (26, 27) was created for CTG interpretation and analysis, incorporating FIGO 2015 guidelines, in its last version (2). The traces were analyzed using the Omniview SisPorto 4.1 at a sampling frequency of 4 Hz. Four basic CTG features were extracted from the SisPorto analysis:

1. Basal line mean level of the most horizontal and less oscillatory FHR segments, in the absence of fetal movements and uterine contractions, associated with periods of fetal rest;
2. Abnormal short-term variability (STV)—percentage of subsequent FHR signals differing less than 1 bpm;
3. Abnormal long-term variability (LTV)—percentage of FHR signals with a difference between the minimum and maximum values in a 1 min window lower than 5 bpm;
4. Saltatory Index—>35% signals outside a filtered band exceeding 25 bpm in last 30, 20, 10, and 5 min.

### 2.3. Fragmentation Analysis

Considering the time series *X* = {*X*_1_, *X*_2_, ..., *X*_*N*_}, where *X*_*i*_ represents the time of occurrence of the fetal normal sinus beat in the instance *i*, the differences between consecutive beats were defined as Δ*X*_*i*_ = *X*_*i*_−*X*_*i*−1_.

#### 2.3.1. Fragmentation Indices

From these time series, four fragmentation indices were computed as proposed by Costa et al. (20). Briefly

1. PIP-Percentage of inflection points.For the calculation of this index, *X*_*i*_ was considered an inflection point when the condition Δ*X*_i_*Δ*X*_*i*+1_ ≤ 0 was verified. Furthermore, the points considered could also be divided into two different types of inflection points:
  1.1 PIP*hard*—when Δ*X*_i_*Δ*X*_*i*+1_ <0.
  1.2 PIP*soft*—when Δ*X*_i_*Δ*X*_*i*+1_ = 0.
  These points represent the instants in which either the acceleration sign inverts (PIP *hard*) or it changes to or from zero (PIP *soft*).
2. IALS-Inverse of the average length of acceleration and deceleration segments.
  An acceleration or a deceleration can be defined as a segment between two consecutive inflection points in the fetal heart rate. For each segment, if the difference between two beats is negative (Δ*X*_*i*_ <0) it is considered a deceleration. On the other hand, if the difference is positive it is considered a acceleration (Δ*X*_*i*_>0). However, there can also be cases in which Δ*X*_*i*_ = 0, meaning that it is not either an acceleration or a deceleration. For the computation of this parameter, these segments were disregarded.
  The size of each acceleration/deceleration is given by the number of points belonging to *X*_*i*_ within that segment.
3. PSS-Percentage of short segments.
  A short segment is considered short if it contains <3 intervals. The PSS was calculated as the complement of the percentage of points in segments of accelerations or decelerations with three or more intervals. It translates to groups of three or more Δ*X*_*i*_ points with the same negative or positive signals in a row.
4. PAS-Percentage of alternating segments.
  An alternating segment is a sequence of at least four Δ*X*_*i*_ points where the sign differs in every single beat. The PAS measure is looking for the percentage of patterns of accelerations (acc) and deceleration (dec) like “acc-dec-acc-dec” or “dec-acc-dec-acc.”

The approach is based on the assumption that the higher the signal's alternation, the more fragmented the time series translates into higher indices.

**Figure 1:**
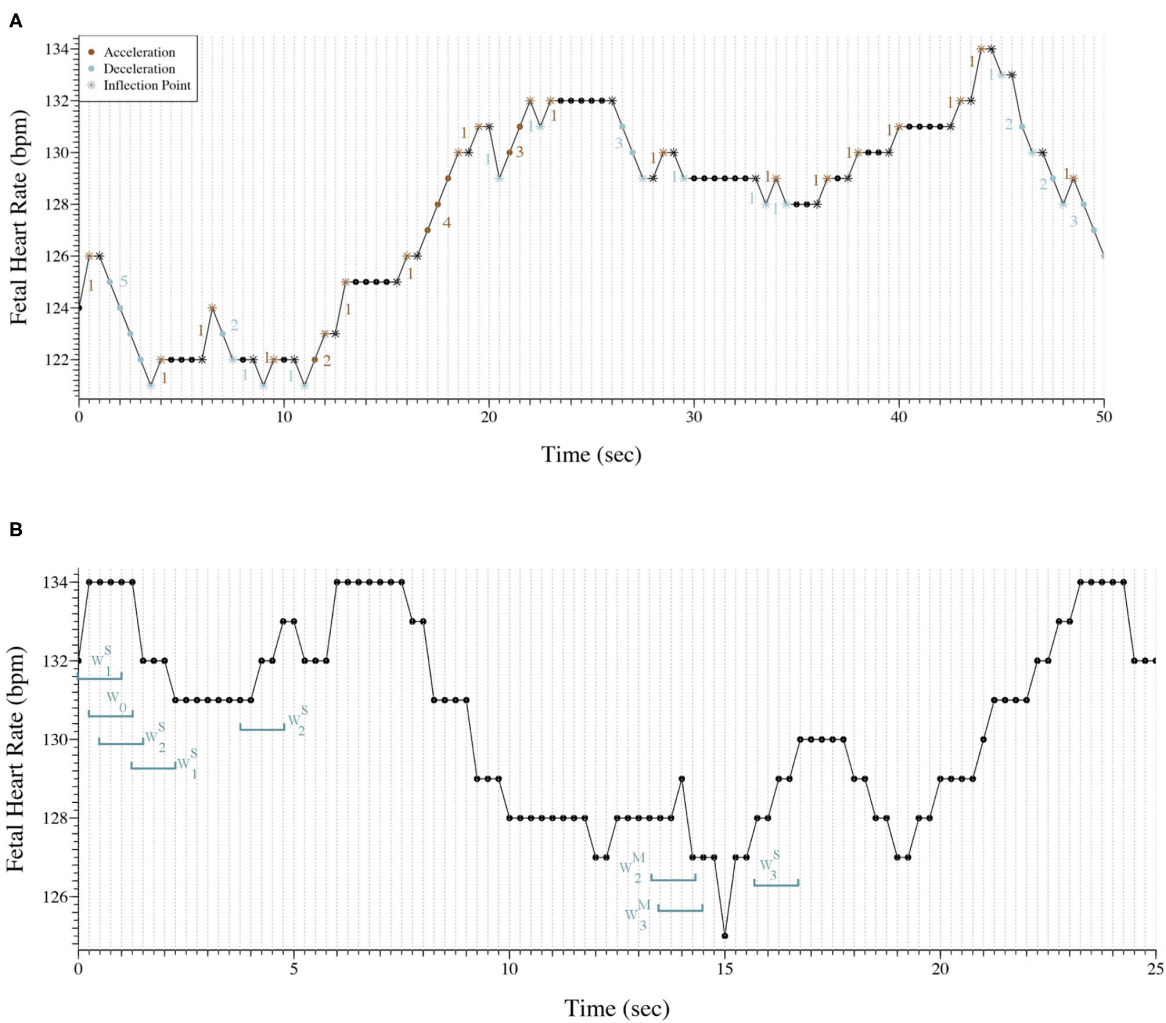
**(A)** Representation of 50 s of a fetal heart rate time series sampled at 2 Hz. The trace presents 53 inflection points (*) and there are 33 segments between inflections points that are accelerations or decelerations. $PIP=\frac{53}{101}\approx 53\%$; $PIP\text{hard}=\frac{11}{101}\approx 11\%$; $PIP\text{soft}=\frac{42}{101}\approx 42\%$; *IALS*≈66%; *PSS*≈82%; *PAS* = 0. **(B)** Representation of 25 s of a fetal heart rate time series sampled at 4 Hz with the classification of eight words as an example.

Figure 1A, shows 50 s (101 points) of a FHR trace sampled at 2 Hz. The trace presents 53 inflection points in which 11 are classified as *hard*. The PIP indices for this trace are the following: $PIP=\frac{53}{101}\approx 53\%$; $PIP\text{hard}=\frac{11}{101}\approx 11\%$; $PIP\text{soft}=\frac{42}{101}\approx 42\%$. Also, there are 33 segments between inflection points that are accelerations or decelerations, therefore $IALS={\left[\frac{\begin{array}{c} 1+5+1+1+2+1+1+1+2+1+1+4+1+1+\\ 3+1+1+3+1+1+1+1+1+1+1+1+1+1\\ +1+2+2+1+3 \end{array}}{33}\right]}^{-1}\approx 66\%$. At last, $PSS=1-\frac{5+4+3+3+3}{101}=1-\frac{18}{101}\approx 82\%$; *PAS* = 0.

#### 2.3.2. Symbolic Fragmentation Indices

The vector Δ*X*_*i*_ = *X*_*i*_−*X*_*i*−1_ was mapped to a ternary symbolic sequence as follows: *s*_*i*_ = 0 if Δ*X*_*i*_=0, *s*_*i*_ = 1 if Δ*X*_*i*_>0, and *s*_*i*_ = 2 if Δ*X*_*i*_ <0. That means that an acceleration corresponded to the number 2, a deceleration corresponded to the number 1, and, in the case of two equal consecutive intervals, it corresponded to 0. Considering i the index of the ternary symbolic sequence, short-terms with 4 elements named “words” (w) were build as follows *w*_*i*_ = {*s*_*i*_, *s*_*i*+1_, ..., *s*_*i*+4−1_}.

Transitions from symbol “1” to “2” or vice versa, were termed hard (H) inflection points. Transitions to or from zero were termed soft (S) inflection points. Word groups with only hard, only soft, and a combination of hard and soft inflection points (mixed) were, respectively, labeled $W_{j}^{h}$, $W_{j}^{s}$, and $W_{j}^{m}$, j indicates the number of inflection points. To calculate each word's percentage, we use the total number of each word as denominators.

Figure 1B exhibits 25 s (101 points) of a FHR trace sampled at 4 Hz. Eight words of length four were selected and classified to better illustrate the symbolic fragmentation indices analysis.

### 2.4. Statistical Analysis

The normality of the fragmentation indices in both groups (normal vs. pathological) was verified by observing the histograms and Q-Q graphs. Since almost all indices' distribution was skewed, values were described with the median and interquartile interval [first quartile-Q1, third quartile-Q3]. The Mann-Whitney *U*-test was used to compare the indices in each of the two groups. Cliff's delta was computed to estimate the effect size. Small effect size was considered when Cliff's delta was between 0.15 and 0.33, medium effect size if Cliff's delta was between 0.33 and 0.47 and large effect size when Cliff's delta was higher than 0.47. The correlation between the matching time series' computed indices was calculated using the Spearman correlation coefficients. For descriptive and inference statistics, SPSS Statistics (v.25; IBM SPSS, Chicago, IL) and R software (28) were used. For all statistical tests, it was used a significance level of 0.05.

## 3. Results

When analyzing the original signals sampled at 4 Hz, from the basic CTG features, only the saltatory index showed significantly higher values in the tracings of the group of fetuses with acidemia compared to those of normal ones. Using the fragmentation measures, we found values of PIP, IASL, and PSS values significantly lower in the tracings of the pathological group (Table 2). The higher PIP in the traces from healthy fetuses represent more inflection points, this is, they oscillate more. The lower value of IASL in the tracings of pathological fetuses means that the size of accelerations or decelerations is higher in that group. In agreement with the previously described results, these traces present less beat-to-beat oscillations. Complementary, PSS as a measure of short segments of three or more beats that are not accelerations or decelerations are also lower in the acidemia group. The results also show that the traces analyzed have a large percentage of two consecutive points of equal value (PIP*soft*), which implies a low value of PAS (mostly zeros). Furthermore, using the symbolic approach in the original traces, the indices $W_{1}^{h}$ and $W_{2}^{s}$ presented significantly higher values in the tracings of the group of fetuses with acidemia compared to those of normal fetuses. The number of words with soft transitions is much higher than both hard and mixed words. These values corroborate the high percentage of two equal consecutive values.

**Table 2:** Comparison of fragmentation indices for both groups using Mann-Whitney *U*-test when analyzing the 4 Hz fetal heart rate time series.

	**Normal**		**Pathologic**			**Cliff's delta effect size**	**Spearman correlation**
	**med**	**[Q1–Q3]**	**med**	**[Q1–Q3]**	***P*-Value**		
**4 Hz**							
Basal line	128	[120–138]	136	[123–142]	0.091	0.17 (s)	
Abnormal STV	46	[39.5–57]	44	[39–55]	0.321	
Abnormal LTV	2	[0–6]	2	[0–5]	0.750	
Saltatory index	79	[48–114]	98	[66.5–134.5]	**0.028**	0.22 (s)	
PIP	97.0	[95.8–97.9]	96.0	[95.0–97.3]	**0.016**	−0.24 (s)	
PIP*hard*	2.5	[1.4–4.2]	3.2	[1.9–4.6]	0.122	
PIP*soft*	94.2	[91.3–96.1]	93.6	[90.9–94.5]	0.056	−0.19 (s)	
IASL	0.92	[0.89–0.94]	0.89	[0.87–0.92]	**0.001**	–0.33 (m)	
PSS	99.4	[98.8–99.8]	99.0	[98.2–99.6 ]	**0.007**	−0.27 (s)	
PAS	0.00	[0.00–0.03]	0.00	[0.00–0.06 ]	0.471	
W_0_	15.7	[8.9–22.2]	17.0	[10.0–22.2]	0.926	
W${}_{1}^{s}$	16.9	[15.1–19.0]	16.7	[14.9–18.4]	0.336	
W${}_{1}^{h}$	0.03	[0.01–0.06]	0.06	[0.03–0.11 ]	**0.003**	0.30 (s)	
W${}_{2}^{s}$	31.0	[28.9–32.9]	32.1	[30.8–35.0 ]	**0.010**	0.26 (s)	
W${}_{2}^{m}$	1.9	[1.3–3.3]	2.5	[1.6–3.6 ]	0.079	0.18 (s)	
W${}_{2}^{h}$	0.01	[0.00–0.01]	0.01	[0.00–0.02 ]	0.277	
W${}_{3}^{s}$	21.6	[16.6–30.5]	20.30	[17.0–24.1]	0.290	
W${}_{3}^{m}$	8.9	[6.3–11.4]	9.6	[7.5–13.10]	0.141	
W${}_{3}^{h}$	0.00	[0.00–0.00]	0.00	[0.00–0.00 ]	0.136	
**2 Hz**							
PIP	76.6	[72.3–82.5]	76.8	[72.2–79.9]	0.316		0.33^**^
PIP*hard*	21.0	[14.8–24.2]	18.6	[14.7– 22.9]	0.262		0.53^**^
PIP*soft*	58.1	[48.4–65.0]	57.2	[51.1–61.6]	0.657		0.21^**^
IASL	0.6	[0.6–0.7]	0.6	[0.6–0.7]	**0.046**	−0.26 (s)	0.39^**^
PSS	79.5	[75.0–85.5]	78.7	[73.1–82.3]	0.091	−0.17 (s)	0.16^*^
PAS	8.8	[4.5–11.2]	7.8	[4.6–9.4 ]	0.074	−0.18 (s)	0.29^**^
W_0_	10.3	[7.8–13.0]	11.6	[9.9–13.4]	**0.026**	0.22 (s)	0.71^**^
W${}_{1}^{s}$	16.0	[13.4–21.1]	18.1	[14.3–21.5]	0.260		0.86^**^
W${}_{1}^{h}$	6.2	[4.3–9.3]	6.7	[5.4–10.6 ]	0.160		0.04
W${}_{2}^{s}$	20.1	[18.6–23.2]	21.2	[19.4–22.8 ]	0.385		0.23^**^
W${}_{2}^{m}$	11.8	[10.7–12.8]	11.0	[10.4–11.8 ]	**0.024**	−0.23 (s)	0.40^**^
W${}_{2}^{h}$	5.9	[3.6–8.2]	5.1	[4.1–7.1 ]	0.632		0.32^**^
W${}_{3}^{s}$	7.4	[6.5–8.3]	6.9	[6.0–8.1]	0.120		−0.44^**^
W${}_{3}^{m}$	16.5	[13.0–18.9]	15.1	[12.5–17.2]	0.077	−0.18 (s)	0.58^**^
W${}_{3}^{h}$	3.1	[1.5–4.0]	2.7	[1.4–3.6 ]	0.194		0.23^**^

*med, median; Q1, first quartile; Q3, third quartile. s, small effect size; STV, short-term variability; LTV, long-term variability;*

* *p < 0.05*,

** *p < 0.001; m, medium effect size*.

*Bold P-value represent the values lower than 0.05*.

When the down-sampled 2 Hz signals are analyzed, PAS values increase while PSS values decrease, indicating less repetitive values in the 2 Hz traces than the 4 Hz ones (Table 2). However, the hypoxia classification power reduces for all indices. Moreover, the symbolic indices applied to these 2 Hz traces show significantly higher values of *W*_0_ in traces from pathological fetuses, meaning that the traces of pathological fetuses present more patterns of four repeated values than the healthy fetus. Also, we found significantly lower values of $W_{2}^{m}$, meaning that patterns with two inflection points are more frequent in traces from healthy fetuses than pathological ones.

In Table 2, the Spearman correlation coefficients between the indices obtained when using the 4 Hz and the matching 2 Hz time series are presented. The achieved correlations are moderate for the fragmentation, being higher for the PIP*hard* index (*r* = 0.53). Notwithstanding, the values obtained with the 2 Hz time series are almost 10 times higher. In the symbolic fragmentation approach, the words *W*_0_, $W_{1}^{s}$, and $W_{3}^{m}$, exhibited higher correlation values (*r* = 0.71, 0.86, and 0.58, respectively). We highlight the no significant correlation found in the $W_{1}^{h}$ index (*r* = 0.04) and the moderate negative correlation obtained in the index $W_{3}^{s}$ (*r* = –0.44).

The Spearman correlations between the SisPorto features, computed using the fetal heart rate traces at 4 Hz, and the fragmentation indices at 4 and 2 Hz are presented in Table 3. The fragmentation indices PIP*hard* and PIP*soft*, computed in the FHR time series at 4 Hz, are moderately correlated with the basal line values. Moreover, the symbolic fragmentation indices presented moderate correlations with the variability indices. Furthermore, when analyzing the FHR time series at 2 Hz, the PIP, the IASL, the PSS and the $W_{1}^{h}$ indices are strongly correlated with the SisPorto variability indices, in particular with the STV. Higher values of the fragmentation indices are correlated with higher values of abnormal STV and LTV. On the other hand, higher values of fragmentation indices represent lower values of Saltatory index.

**Table 3:** Spearman correlation, and corresponding 95% confidence intervals, between Sisporto clinical features, computing using 4 Hz fetal heart rate time series and fragmentation indices for the 4 and 2 Hz fetal heart rate time series.

	**Basal line**	**Abnormal STV**	**Abnormal LTV**	**Saltatory index**
**4 Hz**				
PIP	–0.37 [–0.48; –0.25]	0.22 [0.10; 0.34]	0.17 [0.04; 0.29]	–0.33 [–0.44; –0.21]
PIP*hard*	**0.65 [0.57; 0.73]**	0.29 [0.17; 0.40]	0.29 [0.17; 0.40]	–0.11 [–0.24; 0.01]
PIP*soft*	**–0.63 [–0.70; –0.54]**	–0.11 [–0.23; 0.02]	–0.13 [–0.25; 0.00]	–0.05 [–0.18; 0.07]
IASL	–0.34 [–0.45; –0.23]	0.19 [0.07; 0.31]	0.18 [0.06; 0.30]	–0.38 [–0.48; –0.26]
PSS	–0.07 [–0.20; 0.05]	0.22 [0.10; 0.34]	0.17 [0.05; 0.29]	–0.45 [–0.54; –0.33]
PAS	0.29 [0.17; 0.40]	0.06 [–0.07; 0.18]	0.08 [–0.05; 0.20]	0.04 [–0.08; 0.17]
*W*_0_	0.20 [0.08; 0.32]	**0.59 [0.50; 0.67]**	**0.40 [0.29; 0.51]**	–0.36 [–0.47; –0.24]
$W_{1}^{s}$	–0.05 [–0.17; 0.08]	0.36 [0.24; 0.47]	0.09 [–0.04; 0.21]	–0.33 [–0.44; –0.21]
$W_{1}^{h}$	0.02 [–0.11; 0.14]	–0.26 [–0.37; –0.13]	–0.18 [–0.30; –0.05]	**0.45 [0.34; 0.55]**
$W_{2}^{s}$	0.12 [–0.01; 0.24]	**–0.46 [–0.56; –0.36]**	–0.34 [–0.45; –0.22]	**0.48 [0.37; 0.58]**
$W_{2}^{m}$	**0.73 [0.66; 0.79]**	0.43 [0.32; 0.53]	0.38 [0.26; 0.49]	–0.18 [–0.30; –0.05]
$W_{2}^{h}$	0.13 [0.00; 0.25]	–0.12 [–0.25; 0.00]	–0.10 [–0.22; 0.03]	0.27 [0.15; 0.38]
$W_{3}^{s}$	**–0.65 [–0.72; –0.56]**	**–0.62 [–0.70; –0.53]**	–0.44 [–0.54; –0.33]	0.33 [0.21; 0.44]
$W_{3}^{m}$	**0.49 [0.39; 0.59]**	0.05 [–0.08; 0.17]	0.11 [–0.02; 0.23]	0.01 [–0.11; 0.14]
$W_{3}^{h}$	0.29 [0.17; 0.40]	–0.01 [–0.13; 0.12]	0.01 [–0.12; 0.13]	0.13 [0.01; 0.26]
**2 Hz**				
PIP	0.35 [0.24; 0.46]	**0.86 [0.82; 0.89]**	**0.67 [0.58; 0.74]**	**–0.63 [–0.70; –0.54]**
PIP*hard*	0.02 [–0.11; 0.14]	–0.15 [–0.27; –0.02]	0.00 [–0.12; 0.13]	0.01 [–0.11; 0.14]
PIP*soft*	0.22 [0.09; 0.33]	**0.64 [0.55; 0.71]**	**0.41 [0.30; 0.52]**	**-0.42 [–0.52; –0.31]**
IASL	0.36 [0.24; 0.47]	**0.85 [0.80; 0.88]**	**0.68 [0.60; 0.75]**	**-0.72 [–0.78; –0.65]**
PSS	0.37 [0.25; 0.47]	**0.88 [0.85; 0.91]**	**0.69 [0.60; 0.75]**	**–0.71 [–0.78; –0.64]**
PAS	0.09 [–0.03; 0.22]	0.04 [–0.09; 0.16]	0.12 [0.00; 0.25]	–0.16 [–0.28; –0.03]
*W*_0_	0.20 [0.08; 0.32]	0.22 [0.10; 0.34]	0.15 [0.03; 0.27]	0.06 [–0.07; 0.18]
$W_{1}^{s}$	0.03 [–0.10; 0.15]	0.22 [0.10; 0.34]	0.05 [–0.08; 0.17]	–0.08 [–0.20; 0.05]
$W_{1}^{h}$	–0.30 [–0.42; –0.18]	**–0.80 [–0.85; –0.74]**	**–0.54 [0.50; 0.67]**	**0.60 [0.51; 0.68]**
$W_{2}^{s}$	–0.18[–0.30; –0.05]	0.02 [–0.11; 0.14]	–0.13 [–0.25; –0.01]	–0.01 [–0.14; 0.11]
$W_{2}^{m}$	0.19 [0.07; 0.31]	0.36 [0.24; 0.47]	0.38 [0.26; 0.48]	–0.37 [–0.48; –0.26]
$W_{2}^{h}$	–0.08 [–0.20; 0.05]	–0.36 [–0.47; –0.24]	–0.19 [–0.31; –0.07]	0.18 [0.05; 0.30]
$W_{3}^{s}$	–0.03 [–0.15; 0.10]	0.33 [0.22; 0.44]	0.10 [–0.02; 0.23]	–0.32 [–0.43; –0.20]
$W_{3}^{m}$	0.15 [0.02; 0.27]	0.30 [0.18; 0.41]	0.35 [0.23; 0.46]	–0.32 [–0.43; –0.20]
$W_{3}^{h}$	0.09 [–0.03; 0.22]	0.02 [–0.11; 0.14]	0.13 [0.00; 0.25]	–0.10 [–0.22; 0.02]

*STV, Short-term variability; LTV, Long-term variability; bold correlation values represent moderate to high correlations |r|>0.40*.

## 4. Discussion

The recently proposed fragmentation measures analyze the short-term fluctuations in cardiac beat-to-beat intervals. The novelty of this study is to apply this new fragmentation approach to FHR signals. When applied to FHR, we found that the indices seem to detect pathological patterns in FHR tracings, such as those from acidemic fetuses. In fact, we observed that five of the fragmentation indices, the PIP, IASL, PSS, $W_{1}^{h}$, and the $W_{2}^{s}$, successfully distinguished the traces of fetuses with acidemia from normal fetuses. These indices also have the advantage of being suitable and straightforward to apply in real-time analysis.

Both fragmentation approaches, the original and the symbolic one, analyze the signal taking into account consecutive accelerations or decelerations, ignoring their magnitude. This procedure relates to the analysis of the STV of the signal. The STV characterizing the beat-to-beat variability is, on average, 2 or 3 bpm and reduced if one or less (1). LTV represents broad-based swings in fetal heart rate, or “waviness,” occurring up to several times a minute—it is normal in a bandwidth amplitude of 5–25 bpm. One form of long-term variability of particular significance is a fetal heart “acceleration.” These usually occur in response to fetal movement, and are 15 bpm above the baseline or more, lasting 15 s or longer (12). The presence of fetal accelerations is reassuring that the fetus is healthy and tolerating the intra-uterine environment well. Its absence during labor is of no significance. The STV has been studied as one of the predictors of fetal wellbeing in labor, measuring the dynamic interaction between the fetal sympathetic and parasympathetic nervous systems and its effects on fetal cardiovascular activity (29). As the parasympathetic nervous system is more responsible for variations in STV, it might be reduced in central nervous system hypoxia/ acidosis (23). As described before, if hypoxia is sustained and increases in severity, it leads to the loss of long–term variability (22)—resulting in a global decrease of sympathetic and parasympathetic activity. On the other hand, it has been shown that fetal hypoxia's early effects increased short and long term variability (23). The saltatory pattern or increased variability pattern is described as a bandwidth value exceeding 25 bpm lasting more than 30 min (12)—the pathophysiology of this pattern is incompletely understood, but it may be seen linked with recurrent decelerations, when hypoxia/acidosis evolves very rapidly. It is presumed to be caused by fetal autonomic instability/hyperactive autonomic system (30).

Additionally, in the FHR analyzed, the number of consecutive points with the same value is high. The rationale for this finding may be related to the nature of these indices and the redundant values of FHR signals obtained at 4 Hz. A normal fetal heart rate will be expected to vary from 110 to 160 beats-per-minute in an intrapartum setting, corresponding to frequencies between 1.8 and 2.7 Hz. Therefore, we decided to study the indices applied to 2 Hz downsampled time series. Our results verify this theory once correlations were found between the fragmentation indices and the SisPorto variability features. In fact, we found stronger correlations between the fragmentation indices and the SisPorto variability features, especially with the abnormal STV, when considering the 2 Hz time series. In contrast, no correlations were observed if one analyzed the 4 Hz FHR time series.

Our results were consistent with the results observed in previous studies (17–19). We encountered a moderate correlation between the indices computed in the time series with the different sampling rates, but their values varied greatly. In fact, in Romagnoli et al. (19) the authors used the same database used in this paper and considered the 2 Hz acquisition the ideal for their analysis. The results obtained with the 2 Hz seem to be more physiological, but its ability to distinguish traces from acidemic fetuses appears to decrease. A reason for the obtained results might be that when the FHR signal is sampled at 4 Hz when there is no new beat within 0.25 s, a repetition of FHR values will occur, suggesting that 2 Hz sampling may be the best solution. Figure 1B is good example of repetitive values in the 4 Hz time series. Almost always, there are at least two consecutive points with the same values. Although, in tachycardia where the FHR increases, more common in the pathologic cases, some information might be lost when using 2 Hz acquisition (17–19).

Furthermore, we believe that the symbolic fragmentation outcomes can be improved. The percentage of non-inflection points might be one of the conditions to be further studied, as well as the length of the word chosen. In the original paper, the choice of words of size four was based on the coupling between the cardio-respiratory systems in adults. In FHR, other sizes should be probed to capture the correct dynamic.

The reduced number of pathologic fetuses limited the number of indices to probe in the logistic regression. Future studies should test the combination of these indexes with others used to detect fetal hypoxia to improve the power of discrimination.

## 5. Conclusion

In this exploratory work, the recently proposed fragmentation measures emerge to detect pathological patterns in FHR tracings. Both fragmentation approaches have the advantage of being quick and straightforward to calculate what may be essential for using these measures in real-time settings. In addition, these measures are related to the Sisporto variability indices, especially with the short-term variability of the signal. The question of the ideal sampling frequency for the FHR time series was raised. If, on one hand, the 2 Hz time series avoid multiple duplicated values, it might lose relevant information when the FHR arises in accelerations and tachycardia episodes. On the other hand, this duality might affect the discriminant power of the indices. Future studies should test the combination of these indexes with others used successfully to detect fetal hypoxia to improve the power of discrimination in a larger dataset. This may contribute to developing new computerized algorithms that may improve CTG diagnostic ability to detect fetal hypoxia/ acidosis.

## Data Availability Statement

Publicly available datasets were analyzed in this study. This data can be found here: https://physionet.org/content/ctu-uhb-ctgdb/1.0.0/.

## Ethics Statement

Ethical review and approval was not required for the study on human participants in accordance with the local legislation and institutional requirements.

## Author Contributions

MC and MX as Biomedical students analyzed the data, while TH closely supervised the work. TH wrote the paper, conceived and designed the study. IN wrote, edited, and reviewed the clinical part of the manuscript. All the authors contributed to the manuscript draft, revised, read, and approved the final version of the manuscript.

## Conflict of Interest

The authors declare that the research was conducted in the absence of any commercial or financial relationships that could be construed as a potential conflict of interest.

## Publisher's Note

All claims expressed in this article are solely those of the authors and do not necessarily represent those of their affiliated organizations, or those of the publisher, the editors and the reviewers. Any product that may be evaluated in this article, or claim that may be made by its manufacturer, is not guaranteed or endorsed by the publisher.
